# Time Series Data Generation Method with High Reliability Based on ACGAN

**DOI:** 10.3390/e27020111

**Published:** 2025-01-23

**Authors:** Fang Liu, Yuxin Li, Yuanfang Zheng

**Affiliations:** School of Information Science and Engineering, Shenyang Ligong University, Shenyang 110159, China; liyx524@163.com (Y.L.); zhengyuanfang623@163.com (Y.Z.)

**Keywords:** small sample problem, generative adversarial network, long short-term memory network, time series data generation

## Abstract

In the process of big data processing, especially in fields like industrial fault diagnosis, there is often the issue of small sample sizes. The data generation method based on Generative Adversarial Networks(GANs) is an effective way to solve this problem. Most of the existing data generation methods do not consider temporal characteristics in order to reduce complexity. This can lead to insufficient feature extraction capability. At the same time, there is a high degree of overlap between the generated data due to the low category differentiation of the real data. This leads to a lower level of category differentiation and reliability of the generated data. To address these issues, a time series data generation method with High Reliability based on the ACGAN (HR-ACGAN) is proposed, applied to the field of industrial fault diagnosis. First, a Bi-directional Long Short-Term Memory (Bi-LSTM) network layer is introduced into the discriminator.It can fully learn the temporal characteristics of the time series data and avoid the insufficient feature extraction capability. Further, an improved training objective function is designed in the generator to avoid high overlap of generated data and enhance the reliability of generated data. Finally, two representative datasets from the industrial fault domain were selected to conduct a simulation analysis of the proposed method. The experimental results show that the proposed method can generate data with high similarity. The dataset expanded with the generated data achieves high classification accuracy, effectively mitigating the issue of dataset imbalance. The proposed HR-ACGAN method can provide effective technical support for practical applications such as fault diagnosis.

## 1. Introduction

With the rapid development of information technology, the modeling methods based on machine learning and deep learning have become important research directions in the field of big data processing [1,2,3]. In the process of big data processing, there is often the issue of a small sample problem [4]. For example, in the medical field, it is difficult to obtain enough data for accurate modeling to provide decision aids due to privacy issues or the problem of fewer cases. In the industrial field [5,6,7], fault data is much less than normal data. Moreover, imbalanced datasets can lead to poor fault diagnosis performance. This can easily lead to huge losses and disasters. In the electric power field [8,9,10], there can be an extreme lack of data on abnormal electricity use and theft, and an uneven distribution of positive and negative class samples. Therefore, it is difficult to have a good performance in the detection model. The above examples show that the effectiveness of models in different domains is often limited due to data scarcity or category imbalance. Adequate training samples and a relatively balanced sample distribution are key factors to safeguarding the learning ability and generalisation of models [11,12]. Based on this background, diffusion modeling has been proposed and widely used in the field of data generation such as image and audio. The core idea is to recover data through a stepwise noise addition and reverse denoising process based on a Markov process. For example, in ref. [13], a diffusion model-based time series prediction method was proposed, aiming to address the challenges faced by traditional machine learning methods when dealing with irregularly spaced time series data. However, the computational cost of the diffusion model was high because the model needed to perform a large number of iterative steps to generate data gradually. In contrast, generative adversarial networks (GANs) have a significant advantage in terms of computational cost and require relatively lower costs. In 2014, Goodfellow et al. [14] proposed GANs. The generative adversarial networks have been widely used as an effective generative model for solving small sample problems. GANs can generate synthetic data that are very close to the real data distribution by training a generator and a discriminator to perform adversarial learning. This synthetic data can be used to expand the training set. This can be effective in solving small sample problems and improving the generalization ability of the model [15,16,17].

As the demand for generating diversified and high-quality data increases, GAN models need to be improved. Researchers have improved them in the following two main ways: one is to optimize their structure for improved performance, and the other is to combine a GAN with other models to enhance their generative ability and applicability.

The first improvement optimizes its structure to enhance performance. To compensate for the lack of conditional control in the generation process of the original GAN model, Mirza et al. [18] proposed the conditional generative adversarial network (CGAN). The model generates data with specified classification labels by introducing conditional information into the generator and discriminator. In order to further improve the generation quality of generative adversarial networks and the classification performance in practical applications, in 2017, ref. [19] proposed the auxiliary classifier generative adversarial network (ACGAN) model. The model takes random noise and classification labels as input to the generator and uses the discriminator to discriminate between the classification labels. The ACGAN model not only improves classification performance but also guides the generation of data with specified classification labels. To improve model stability, the WGAN with Gradient Penalty (WGAN-GP) model [20] was proposed. The model introduces a penalty term instead of weight clipping to avoid the mode collapse issue. Dan et al. proposed the anomaly detection methods, GAN-AD [21] and MAD-GAN [22], based on generative adversarial networks in 2019. Feature distributions of a multivariate time series are captured using recurrent neural networks. Residuals between the reconstructed data and the actual samples from the GAN-trained discriminator and generator are calculated to detect possible anomalies in complex datasets.

In addition to performance improvements, many GAN-based hybrid models have been proposed in recent years, with the aim of enhancing generation quality and applicability. Radford et al. [23] proposed the deep convolutional generative adversarial network (DCGAN). This model combines convolutional neural networks (CNNs) with a GAN and uses CNN layers as the network structure of the GAN. This improvement makes the model more practical while improving its stability. Ref. [24] proposed a GRU-based GAN model (GRU-GAN). This model attaches the generator of GAN to the output of GRU to realize the extraction and dimensionality reduction of implicit features, as well as to mine the dependencies between long time sequences.

Although the existing research has optimized GAN models and considered temporal dependencies in data generation, the current methods still struggle to effectively capture data features when handling complex temporal dependencies or long-span sequence data, which limits the model’s performance in practical applications [25]. Refs. [26,27] applied a DCGAN to generate bearing fault data and expand the dataset. This significantly improved the diagnostic performance. Yan et al. [28] introduced a GAN to generate data during the fault diagnosis of water chillers. The results showed that this method could perform high-precision fault diagnosis on small sample data. However, these two methods do not sufficiently consider the time dependency, resulting in insufficient model feature extraction capability.

Furthermore, although GAN models have become a research focus in fields such as fault diagnosis and anomaly detection, most existing data generation methods based on generative adversarial networks (GANs) have yet to fully address the potentially high level of overlap between different categories in the generated data. Such overlap has several negative effects on model training. For example, when category samples overlap, the model tends to overfit features in the overlapping regions, making it difficult to learn the distinct characteristics of each category. This reduces the model’s generalization ability and ultimately diminishes the quality and practical value of the generated data. Shao et al. [6] constructed a framework based on the ACGAN model by stacking one-dimensional convolution layers to learn the vibration signal dataset of an induction motor and generate the one-dimensional raw data. The generated data could be applied as enhanced data for machine fault diagnosis. Li et al. [29] proposed an improved ACGAN model designed with a new framework. This model improved the compatibility between source and classification by adding an independent classifier. Additionally, spectral normalization is used to constrain the weight parameters of the discriminator to achieve stable training. Zhou et al. [30] used a global optimization scheme to generate fault samples. The impact of the generated samples on the fault diagnosis results was taken into consideration by adding a DNN model as an additional discriminator. During the training process, the generation ability of the generator, the discrimination ability of the discriminator, and the diagnostic ability of the fault diagnosis model were simultaneously improved. Liu et al. [31] established an improved deep feature enhanced GAN model. This model introduced a pull-away function to design a new generator loss function, thereby improving the stability of the model. Additionally, the self-attention mechanism was introduced to improve the learning ability of the model and further enhance the quality of generated data. This method was validated by the bearing dataset from Case Western Reserve University and the electrical locomotive bearing dataset. The experimental results showed that this method can effectively expand imbalanced datasets. However, these methods take into account less variability between the categories of the real data, whereas the generative adversarial network has a larger generative range. This leads to higher overlap between the categories of generated data and lower reliability of the generated data.

In the current research context, there is often the issue of small sample sizes in fields such as healthcare, industry, and power, especially in tasks like fault diagnosis and anomaly detection. Despite some progress made by GAN models, they still have numerous limitations. On one hand, the currently optimized GAN models struggle to fully extract data features, leading to a decline in the quality of generated data. On the other hand, in fields like fault diagnosis and anomaly detection, existing models fail to fully consider the high overlap that may exist between different categories in the generated data, resulting in reduced model generalization ability. Based on these two issues, this paper proposes a high-reliability time series data generation method based on ACGAN (HR-ACGAN), aiming to address the small sample problem in the industrial fault domain. The contributions of the HR-ACGAN method and the solutions to the above two problems are as follows:

1. First, combine the ideas of WGAN-GP to optimize the ACGAN model and improve training stability. Replace the Jensen-Shannon (JS) divergence or Kullback-Leibler (KL) divergence with the Wasserstein distance to measure the distribution difference between generated data and real data. Introduce gradient penalty instead of weight clipping to satisfy the K-Lipschitz continuity condition, avoiding gradient vanishing or explosion while retaining the advantages of the Wasserstein distance;
2. Then, a modified discriminator model is established by incorporating a Bi-LSTM network layer. The Bi-LSTM network, by combining both forward and backward sequence information, can fully exploit the temporal characteristics of time series data, addressing the issue of insufficient data feature extraction in existing GAN models;
3. Finally, the generator’s objective function is improved by adding a center loss term to reduce the overlap between the different categories of the samples and enhance the reliability of the generated data, addressing the issue that existing methods fail to adequately consider the potential high degree of overlap between the categories in the generated data within fields such as fault diagnosis and anomaly detection. This improvement achieves the following two key goals: it reduces intra-class dispersion by bringing samples of the same category closer together, and it preserves inter-class variability to avoid excessive overlap.

## 2. The System Model

**Figure 1:**
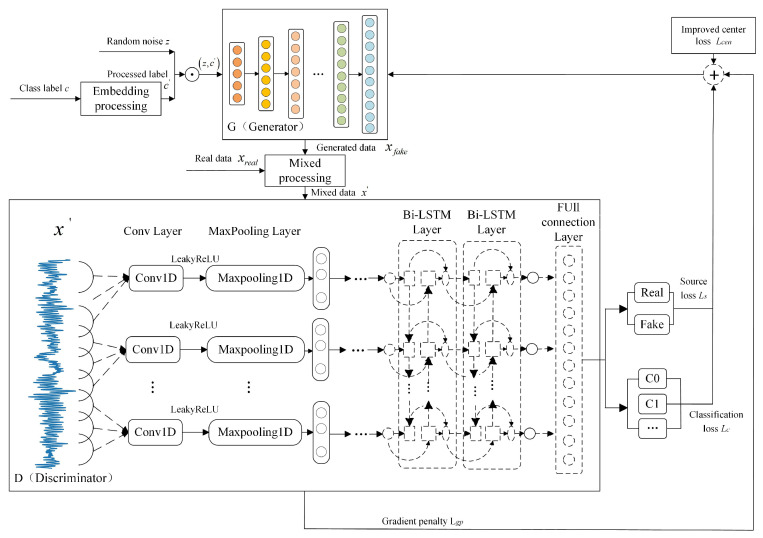
The Overall Architecture of the HR-ACGAN Model.

The overall architecture of the proposed model is shown in Figure 1. Firstly, based on the ACGAN model, a Bi-LSTM network layer is introduced into the discriminator network to construct an improved discriminator model that fully learns the temporal features.The traditional LSTM network can only capture unidirectional temporal dependencies. Therefore, this method introduces Bi-LSTM into the discriminator, which is capable of capturing both forward and backward dependencies in the data sequence. Bi-LSTM consists of two LSTM networks, one handling the forward time series information and the other handling the backward time series information. By incorporating the Bi-LSTM layer, we are able to more comprehensively extract global temporal information from the generated data, thereby effectively improving the quality of the generated data. This improvement is particularly important for practical applications such as industrial fault diagnosis, where the data often exhibits complex temporal dependencies. The introduction of Bi-LSTM helps enhance the performance of data generation tasks in these domains. Secondly, by replacing JS divergence or KL divergence with Wasserstein distance to measure the difference between the generated and real data, and by introducing a penalty term into the true-false discrimination loss, an improved generative model is proposed to enhance the stability of the model. Finally, an improved objective function is designed for the generator to reduce intra-class distances in the generated data, minimize overlap between samples from different categories, and improve the reliability of the generated data. In summary, compared with existing LSTM-based GAN models, the method proposed in this paper performs better in the generation and classification of complex time-series data and exhibits higher stability. The detailed theoretical derivation of the HR-ACGAN method is as follows.

First, the categorical label *c* is processed through an embedding layer to obtain an embedding vector $c^{\prime }$ with the same dimension as the number of categories. The embedding layer is a special layer in the neural networks, primarily used to handle discrete categorical data. Its core function is to map discrete data into a continuous vector space, thereby enhancing the semantic representation of the labels. Using the ACGAN model as the basic framework, a generator model based on a one-dimensional convolutional neural network is established for data generation. The random noise *z* and class label $c^{\prime }$ are input into the generator model G(•), and the generated data $x_{fack}$ can be obtained by Equation (1).

(1) $$x_{fake}=G\left(z,c^{\prime }\right)$$

Then, the generated data $x_{fake}$ and the real data $x_{real}$ are mixed, that is, $x_{fake}$ and $x_{real}$ are concatenated according to each data. The mixed data $x^{\prime }$ is obtained by Equation (2).

(2) $$x^{\prime }=mix\left(x_{real},x_{fake}\right)$$

The mixed data $x^{\prime }$ is fed into the improved discriminator *D*. In the design process of the discriminator structure, in order to make the model more stable, the WGAN-GP idea is therefore combined. The Wasserstein distance is used to measure the similarity between the generated data and the real data, and a penalty term is introduced in the true-false discriminant loss. Considering the extraction of the temporal characteristics of the time series data, an existing Bi-directional Long Short-Term Memory (Bi-LSTM) network considering bi-directional state information is selected for the adaptive combination of the discriminator model structure. After inputting the mixed data $x^{\prime }$ into the discriminator, the source output and classification output can be obtained through the output layer.

After training the discriminator network, the trained discriminator model is used to measure the loss of the generator and guide the training of the generator. In order to solve the problem of low reliability of the generated data due to high overlap, the central loss term is added to the training objective function of the generator.

Then, the proposed HR-ACGAN model is alternately trained by using the objective functions of the discriminator and the generator, respectively, until the model reaches nash equilibrium. By this point, the discriminator has a high discriminating accuracy for both the real and generated data. This ensures that the generated data closely matches the distribution of the real data. It also has a high discriminant accuracy, ensuring that the generated data has a high quality.

Finally, after training the model, the generator is used to generate highly reliable data with specified classification labels according to Equation (1).

## 3. Introduction to Algorithms

### 3.1. Discriminator Model

The mixed data $x^{\prime }$ is fed into the discriminator *D*. In the discriminator, Wasserstein distance is introduced to replace JS or KL divergence for measuring the similarity between generated data and real data. The inclusion of the Wasserstein distance helps improve the convergence speed of the model, avoids the vanishing gradient problem, and further enhances the performance of the model. The formula for the Wasserstein distance is shown in Equation (3):

(3) $$W\left(P_{r},P_{g}\right)=\underset{\gamma \in \Pi (P_{r},P_{g})}{\inf}E_{(x,y)∼\gamma }\left[∥x-y∥\right]$$

where $P_{r}$ is the distribution of real data, $P_{g}$ is the distribution of generated data. $\Pi (P_{r},P_{g})$ is the set of all possible joint distributions.

On a technical level, when introducing the Wasserstein distance into ACGAN, there is no need to use a Sigmoid activation function in the final layer to generate discrimination probabilities. Instead, the output in the real-number space is directly used to measure the Wasserstein distance between the generated data distribution $P_{g}$ and the real data distribution $P_{r}$. By removing the Sigmoid function, the output of the discriminator is no longer constrained to the probability space, allowing it to replace the JS divergence or KL divergence used in traditional GANs, thereby improving the model’s stability.

Since the Wasserstein distance needs to satisfy the K-Lipschitz continuity condition, a gradient penalty term is introduced to ensure that the gradient does not exceed K by adding an additional loss term. Gradient penalty can be calculated by using Equation (4).

(4) $$L_{gp}=\lambda E_{x_{r}∼P_{x_{r}}}\left[{\left(∥\nabla_{x_{r}}D\left(x_{r}\right){∥}_{2}-1\right)}^{2}\right]$$

where $x_{r}$ represents the random linear interpolation between $x_{fake}$ and $x_{real}$, which can be expressed as $x_{r}=\varepsilon x_{real}+\left(1-\varepsilon \right)$$x_{fake}$, ε∼*U*[0,1], *U* represents a uniform distribution; λ represents the penalty coefficient, ∥•∥ represents L2-norm, ∇ represents the gradient calculation, D(•) represents the discriminator model. Therefore, Equation (4) represents the expectation of *E* with respect to the distribution of $x_{r}$ determined by the above linear interpolation process.

Then, the gradient penalty is added to the discriminator loss function of the original ACGAN model. The source loss function $L_{s}$ and classification loss function $L_{c}$ of the discriminator are shown in Equation (5) and Equation (6), respectively.

(5) $$L_{s}=-E_{x∼P_{\mathrm{data}}}\left[\log D\left(x\right)\right]-E_{z∼P_{z}}\left[\log\left(1-D\left(G\left(z\right)\right)\right)\right]+\lambda E_{x_{r}∼P_{x_{r}}}\left[{\left(∥\nabla_{x_{r}}D\left(x_{r}\right){∥}_{2}-1\right)}^{2}\right]$$

(6) $$L_{c}=-E_{c∼P_{\mathrm{data}\;}}\left[\log D\left(c\right)\right]-E_{c∼P_{z}}\left[\log\left(1-D\left(G\left(c\right)\right)\right)\right]$$

The task of the discriminator is to identify the source of data as accurately as possible while classifying as accurately as possible, so the loss function of the discriminator is $L_{c}+L_{s}$. Specifically, in Equation (5), the distribution of the expectation *E* is as follows. The first term is to calculate the expectation of *x* sampled from the real data distribution $P_{\mathrm{data}}$, the second term is to calculate the expectation of *z* sampled from the noise distribution $P_{z}$, and the third term is the expectation with respect to the distribution of the interpolated samples $P_{x_{r}}$. In Equation (6), the expectations *E* are calculated for *c* sampled from the real data distribution $P_{\mathrm{data}}$ and *c* sampled from the noise distribution $P_{z}$.

When dealing with temporal data or complex feature generation tasks, the design of the discriminator needs to fully consider the temporal characteristics and the ability to extract global patterns. Therefore, this method introduces a Bi-LSTM network into the discriminator to fully exploit the temporal characteristics. Compared with the ordinary LSTM, Bi-LSTM can capture both forward and backward temporal dependencies to extract more complete features, while LSTM only captures unidirectional forward dependencies, which may ignore the backward information and lead to incomplete features. Compared with GRU, although its simpler structure and fewer parameters give it an advantage on small-scale data, Bi-LSTM usually performs better considering the complexity of the data and the need for long-time dependency modeling. Compared with a transformer, although it has some advantages in long dependency processing, the high computational complexity and resource demand for large-scale data limit its applicability. After comprehensive consideration, Bi-LSTM was selected as the temporal feature extraction model in this paper.

**Figure 2:**
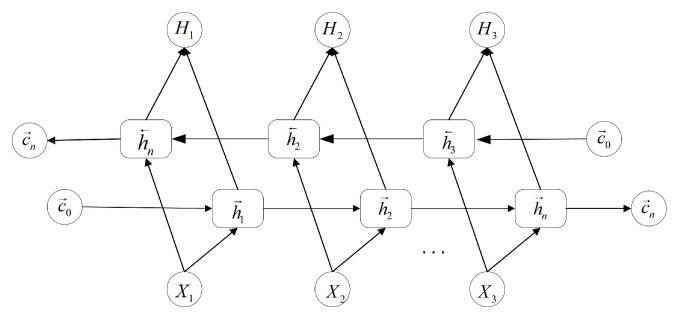
Structure Diagram of Bi-LSTM Network.

By integrating the Bi-LSTM network, the discriminator model is able to fully utilize the information of the entire input sequence to enhance the discriminative ability of the data, which not only enhances the ability of the generator to produce high-quality samples but also significantly improves the classification performance of the discriminator. The Bi-LSTM network structure is shown in Figure 2.

**Figure 3:**
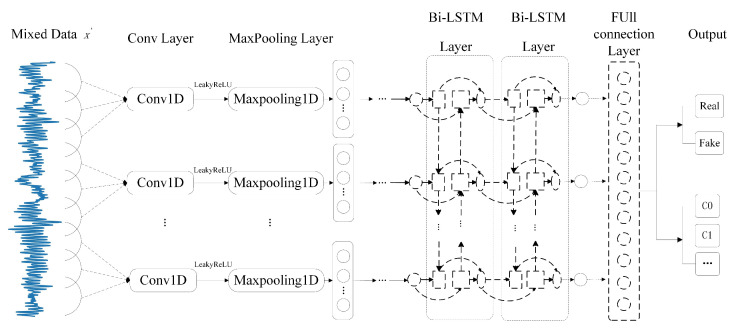
Structural Diagram of the Improved Discriminator Networks.

Next, the Bi-LSTM network mentioned above and convolutional neural networks (CNNs) are introduced into the discriminator network structure to establish an improved discriminator model, and the structural diagram of the improved discriminator network is shown in Figure 3. After entering the mixed data $x^{\prime }$ into the discriminator, the feature information of $x^{\prime }$ is initially extracted through the convolution and pooling layers. Then, the temporal information of the data is extracted through the Bi-LSTM layer. The hidden layer information passed through the Bi-LSTM layer is input into the fully connected layer to integrate hidden layer information into a one-dimensional vector, which can reduce the dimension of the hidden layer information and make it closer to the output. Finally, the source output and classification output can be obtained through the output layer.

### 3.2. Generator Model

After training the discriminator networks, the trained discriminator model is used to measure the loss of the generator and guide the training of the generator. The task of the generator is to classify correctly while making the discriminator classify the generated data as real. Therefore, the loss function $L_{G}$ of the generator is shown in Equation (7).

(7) $$L_{G}=E_{z∼P_{z}}\left[\log\left(1-D\left(G\left(z\right)\right)\right)\right]-E_{c∼P_{z}}[\log\left(1-D\left(G\left(c\right)\right)\right)$$

Next, we improve the generator’s objective function by introducing a central loss term. Traditional methods often lack effective constraints on the distribution range of the generated data. This results in overly broad distributions across the categories, with significant overlap between the samples of different categories.

To address this issue, we modify the central loss term into a dynamic center loss that evolves during the GAN’s iterative process. Specifically, the feature centroids of each category are calculated, and the center loss term is added to the generator’s objective function. This loss term constrains the generated data to be more concentrated around the centroids of their respective categories.

If each training sample has *k* features, the label of the *i*-th class is $y_{i}$, the number of samples with label $y_{i}$ is *N*, the feature center of the *k*-th feature is $c_{k}$, the *k*-th feature of the *j*-th data is $x_{j}^{k}$. The center $c_{y_{i}}$ of the samples with label $y_{i}$ can be obtained from Equation (8).

(8) $$c_{y_{i}}=\left(c_{1},c_{2},\ldots ,c_{k}\right)=\left(\frac{1}{N}\sum_{j=1}^{N}x_{j}^{1},\frac{1}{N}\sum_{j=1}^{N}x_{j}^{2},\ldots ,\frac{1}{N}\sum_{j=1}^{N}x_{j}^{k}\right)$$

Then, the static center loss $L_{cen}$ can be calculated according to $c_{y_{i}}$ as shown in Equation (9). Here, $L_{cen}$ represents the center loss term.

(9) $$L_{cen}=\frac{1}{2}\sum_{i=1}^{N}{∥x_{i}-c_{y_{i}}∥}_{2}^{2}$$

At this point, when calculating the center loss term, $c_{yi}$ is fixed by Equation (9). This leads to a central loss term that makes the generation range of each category too fixed, which does not correspond to the distribution of real data. Therefore, the center loss term is modified, and the modified dynamic center of each type of real data is shown in Equation (10). In this case, the center point gradually shifts toward the new generated features based on the distribution of the generated data, with the update step size controlled by the hyperparameter β.

(10) $$\begin{array}{cc} c_{yi} & =(c_{1},c_{2},\ldots ,c_{k})\\ \; & =\left(c_{1}^{t}-\beta \left(c_{1}^{t-1}-g_{1}^{t-1}\right),c_{2}^{t}-\beta \left(c_{2}^{t-1}-g_{2}^{t-1}\right),\ldots ,c_{k}^{t}-\beta \left(c_{k}^{t-1}-g_{k}^{t-1}\right)\right) \end{array}$$

where β is the moving step length, $c_{k}^{t}$ represents the feature center of the *k*-th feature after the *t*-th iteration, and $g_{k}^{t-1}$ represents the feature center of the *k*-th feature of the generated data after the *t*-th iteration. By adjusting the value of β, we can balance intra-class compactness and inter-class separability. At each iteration of the generator, the feature center of each type moves towards the feature center of the generated data through the selected moving step length. If the value of β is large, the error of the feature center will cause a greater impact, so the value of β should not be excessively large. Typically, β is set to a relatively small constant value to prevent the center loss from excessively influencing model learning. To ensure the rationality of this choice, a grid search is performed over a series of candidate values, such as 0.1–0.5, combining cross-validation methods to evaluate the parameter values that achieve optimal performance on the validation set. Based on the experimental results, β = 0.1 is determined to be the optimal value. According to the modified $c_{y_{i}}$, the new center loss term can be calculated as shown in Equation (11).

(11) $$L_{cen}=\frac{1}{2}\sum_{i=1}^{N}{∥x_{fake}-c_{y_{i}}∥}_{2}^{2}$$

Introducing the center loss term into the generator’s loss function can gradually optimize the distribution of the generated data during the iteration process, making it more concentrated around the center point of the corresponding class. This reduces the within-class distance between similar samples, decreases the overlap between different classes, and improves the quality of the generated data. At this time, the loss function of the generator with the center loss term added is $L_{G}$, and $L_{G}$ is given by Equation (12).

(12) $$L_{G}=-E_{c∼P_{z}}[\log\left(1-D\left(G\left(c\right)\right)\right)+E_{z∼P_{z}}\left[\log\left(1-D\left(G\left(z\right)\right)\right)\right]+\frac{1}{2}\sum_{i=1}^{N}{∥x_{fake}-c_{y_{i}}∥}_{2}^{2}$$

Adding Bi-LSTM and center loss terms to the generator and discriminator will increase computational overhead to some extent. Specifically, compared to standard LSTM, the bidirectional nature of Bi-LSTM increases the computational cost; however, it enables the model to fully capture the bidirectional features of the time series data, significantly enhancing the expression of temporal characteristics in the generated data, thereby improving the quality of the generated data. Additionally, the inclusion of the center loss term also adds some computational overhead, as it requires the extra computation of the distance between the class centers and the samples. However, this step only involves simple distance calculations and optimization, and its computational cost is relatively minor compared to the overall complexity of the network. In summary, these improvements significantly enhance the accuracy of the classification model, and the value brought by the performance improvement far outweighs the increase in computational cost. Moreover, the data generation process is carried out in an offline environment and does not involve real-time prediction, so the computational cost has little impact on the model’s practical application.

## 4. The Simulation Analysis

### 4.1. Data Preprocessing

To validate the effectiveness of the proposed method in the field of industrial fault diagnosis, this paper selects the Case Western Reserve University (CWRU) rolling bearing dataset as the base dataset for simulation experiments. In addition, to further verify the scalability of the method in different industrial fault scenarios, the Gear Fault Diagnosis (GFD) dataset from the University of Connecticut is added as an extension dataset for validation. Through experiments on these two datasets, the applicability and reliability of the proposed method in industrial fault diagnosis are fully demonstrated.

#### 4.1.1. Basic Dataset

To validate the effectiveness of the proposed method in the field of industrial fault diagnosis, this paper uses the rolling bearing dataset from Case Western Reserve University (CWRU) as the basic dataset. This dataset is one of the standard datasets in the field of fault diagnosis, with recognized authority and objectivity, which can effectively support the reliability of the experimental results. The CWRU fault dataset consists of data collected using acceleration sensors, and the sensor is placed at the drive end (DE) and fan end (FE). The sampling frequency of DE is 1.2 kHz and 4.8 kHz, and that of FE is 1.2 kHz. These data points are collected under the load conditions of 0 hp, 1 hp, 2 hp and 3 hp. The fault states include inner ring fault, outer ring fault and rolling element fault. Each type of fault has three diameters: 0.007 inch, 0.014 inch and 0.021 inch, and the dataset has ten states including the normal state.

Ten types of DE data with a sampling frequency of 12 kHz and load states ranging from 0 hp to 3 hp are selected as the experimental dataset. The selected data undergoes sampling processing. In each load state, 400 data points are randomly selected from the normal condition data, while 100 data points are randomly selected from each type of fault data. Each selected data point is then extended by 1023 data points to achieve a total sampling length of 1024 for each data instance. In total, 5200 data samples are generated, with 1600 samples from normal conditions and 400 samples from each type of fault condition. The description of the selected dataset is shown in Table A1 in Appendix A.

In the data generation process, we fully considered the issue of class imbalance and designed corresponding methods to ensure the balance and effectiveness of the generated data. Specifically, we first extracted a portion of data from the original imbalanced dataset to construct a balanced subset, which was used to train the generation model. During the training of the generation model, the balanced dataset was used, allowing the model to generate a specified number of samples for each class. Subsequently, we extended the generated data back into the original imbalanced dataset, thereby constructing a class-balanced dataset. Finally, the resulting class-balanced dataset was used for subsequent experiments and analysis.

For the purpose of training and testing in the experiment, the selected dataset was divided. Firstly, 100 samples of each category were randomly selected to be used as the test set. Then, different numbers of samples of each category were randomly selected from the remaining data to form the imbalanced sample set of the basic dataset, which was used to simulate the imbalanced situation. Next, 150 pieces of data of each category were randomly selected from the imbalanced sample set of the basic dataset as the training data for the generation model, namely the training data of the basic dataset. The size of the experimental dataset can be found in Table A2 of Appendix A.

#### 4.1.2. Extended Dataset

To further validate the scalability of the model in the field of industrial fault diagnosis, we added the University of Connecticut Gear Fault Dataset (GFD) as an additional dataset. This dataset is a benchmark experimental dataset used for gear fault diagnosis research and was designed and collected by the research team of the Department of Mechanical Engineering at the University of Connecticut. We selected the gear time-domain vibration signal data under nine conditions in this dataset. These nine conditions include healthy, missing teeth, cracks, spalling, and sharpening (five different degrees of sharpening).

Firstly, perform sampling processing on the selected data. Under each condition, randomly select data points from the data, and extend each data point backward by 1023 data points, so as to obtain a sampling length of 1024. Next, in order to conduct training and testing for the experiment, the selected data set is divided. Randomly select 100 samples of each type of data as the test set, and then randomly select a total of 1325 data samples under nine conditions (healthy, missing teeth, cracks, spalling, sharpened, and five different degrees of sharpened), with the numbers being 1500, 120, 230, 160, 150, 100, 105, 140 and 120 respectively, from the remaining data. These samples are used as the imbalanced sample set of the extended data set to simulate the imbalanced situation. Finally, 100 data of each type are randomly selected from the imbalanced sample set of the extended data set as the training data for the generation model, which is the training data of the extended dataset.

### 4.2. Parameter Settings and Training Process

In this experiment, the model framework consists of a generator and a discriminator. The model parameters and network structure are selected according to the principle part. The network parameters are set as follows: the batch size is 128, the learning rate of the generator is 0.0002, the learning rate of the discriminator is 0.0001, and the penalty coefficient λ is 10. When determining the value of the penalty coefficient, we referred to ref. [32] and selected 10 as the penalty coefficient for the experiment. The experimental results show that when the penalty coefficient is 10, the model achieves the best training stability and generated sample quality. The dimension of the input random noise is 100, the weight ratio of true–false loss, classification loss, and penalty coefficient is 1:1:10, and the RMSprop optimizer is used.

The generator is used to generate target time series data. The input of the generator is the composite data of 100-dimensional random noise and labels. The upsampling is used to inflate the data to output a batch of generated data with the same dimensions as the real time series data. The model consists of 1D-CNN layers and Batch Normalization (BN) layers. The ReLU function is selected as the activation function after each 1D-CNN layer, and the Tanh function is selected as the activation function of the last layer. The model structure of the generator is shown in Table A3 in Appendix A.

The discriminator also uses the 1D-CNN layer as the main model framework to judge source and classification. The discriminator uses the judgement result to conduct adversarial training with the generator network model. This makes the generated data of higher quality. In the discriminator, the LeakyRelu function is used as the activation function after each 1D-CNN layer, and the slope is set as 0.2. To solve the overfitting problem of the discriminator, the Dropout layer is introduced, and the parameter p is set as 0.5. Finally, the Bi-LSTM layer is introduced to better adapt to the timing characteristics of the time series data. During the classification discrimination, multi-classification cross entropy is used as the loss function. During the source discrimination, Wasserstein distance is used to measure the distance between the date and generated data. The model structure of discriminator is shown in Table A4 in Appendix A.

The model is trained according to the principle of the proposed method, the network structure, and the parameter settings. The training steps are as follows:

1. The generator generates data. A batch of generated data is obtained by mixing random noise and labels, and inputting it to them into the generator. This batch of generated data is mixed with real data and input to the discriminator;
2. Train the discriminator. According to the loss function of the discriminator and the RMSprop algorithm, the discriminator parameters are updated. Training the discriminator first can accelerate the training process of the model;
3. Train the generator. The parameters of the discriminator are guaranteed to remain unchanged. Update the parameters of the generator according to the generator’s loss function and the RMSprop algorithm;
4. Train alternately. Repeat step (2) to step (4) as one epoch of training. After the end of one epoch of training, start a new epoch of training. When Nash equilibrium or set epoch is reached, the training will be stopped.

### 4.3. Ablation Experiment

To verify the effectiveness of the key modules of HR-ACGAN, this paper designs three variants of HR-ACGAN, which are referred to as HR-ACGAN-woGP, HR-ACGAN-woLSTM, and HR-ACGAN-woCL, respectively. The descriptions of the four models, including HR-ACGAN, are shown as follows:

1. HR-ACGAN-woGP model: This model is obtained by removing the penalty term from HR-ACGAN. In this model, the network no longer performs additional constraint optimization on the distribution differences of the generated data.
2. HR-ACGAN-woLSTM model: This model is obtained by removing the Bi-LSTM network from HR-ACGAN and reverting back to the ordinary ACGAN discriminator structure.
3. HR-ACGAN-woCL model: This model is derived from HR-ACGAN by removing the central loss term. In this model, the network no longer optimizes the distinguishability and consistency of the generated data by imposing constraints on class centroids.
4. HR-ACGAN model: The final model proposed in this paper.

#### 4.3.1. Similarity Analysis

We used the basic dataset and adopted four models to detect the similarity. For the comparative analysis of the similarity of these four models on the basic dataset, two metrics, namely the Euclidean distance and the Wasserstein distance, were employed. The Euclidean distance can be used to measure the similarity between two vectors. The higher the similarity is, the smaller the Euclidean distance will be, as shown in Equation (13). The similarity between two sample sets can be obtained by calculating the average value of the Euclidean distances of each vector within them.

(13) $$d\left(X,Y\right)=\sqrt{\sum_{i=1}^{n}{\left(X_{n}-Y_{n}\right)}^{2}}$$

where *X* and *Y* represent two different datasets, respectively, and *n* represents the number of data in datasets.

In addition to Euclidean distance, Wasserstein distance can also be used to evaluate the quality of generated data. Unlike Euclidean distance, Wasserstein distance is primarily used to measure the difference between two probability distributions, specifically the minimum cost required to transform one distribution into another. If the distribution of the generated data completely overlaps with the distribution of the real data, the Wasserstein distance is zero, as shown in Equation (3).

Since the basic dataset has multiple labels, and the Euclidean distance and the Wasserstein distance will be measured for each label in every model, in order to conduct further comprehensive analysis, we added up the distances of multiple labels, respectively, and then divided them by the number of labels, thus obtaining the average Euclidean distance and Wasserstein distance of the dataset under these four models.

**Figure 4:**
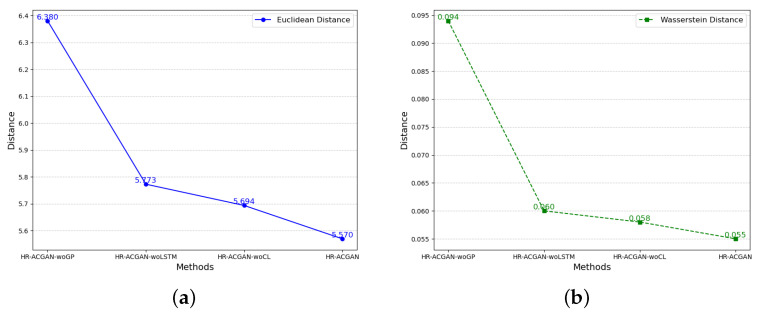
Similarity Analysis Charts of Different Ablation Methods. (**a**) Evaluation Charts of Euclidean Distance for the Generated Data of Different Ablation Methods. (**b**) Evaluation Charts of Wasserstein Distance for the Generated Data of Different Ablation Methods.

As shown in Figure 4, the data generated using the method proposed in this paper is of the best quality, and the penalty term, central loss term, and long short-term memory network (LSTM) set in the model all contribute to the improvement of the model performance. This is specifically reflected in two metrics for measuring the differences between the generated data and the real data, namely the Euclidean distance and the Wasserstein distance.

In terms of the Euclidean distance metric, after adding the penalty term, Long Short-Term Memory network, and central loss term, the Euclidean distance is reduced by 12.7%, 3.52%, and 2.18%, respectively, compared to the original situation. This means that with the addition of these elements, the generated data are closer to the real data in terms of this metric and have become more similar.

In terms of the Wasserstein distance metric, these three elements reduce the Wasserstein distance by 41.49%, 8.33%, and 5.17%, respectively, indicating that they help to narrow the differences between the distribution of the generated data and that of the real data. Among them, the penalty term plays a particularly prominent role in this regard.

#### 4.3.2. Classification Accuracy Analysis

**Figure 5:**
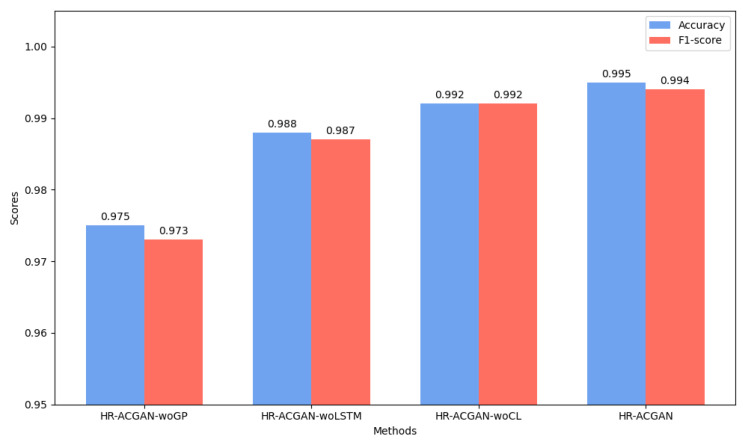
Comparison of the Classification Accuracy and Performance of Different Ablation Models.

We used the basic dataset and adopted the above four models to conduct detection on this basic dataset. The results of the comparative analysis of the classification accuracy of the above four models on the basic dataset are shown in Figure 5. The evaluation metrics for classification accuracy include *accuracy*, *precision*, *recall*, and *F1-score*, as shown in Equations (14)–(17). Accuracy represents the proportion of the number of correctly classified samples to the total number of samples. Precision indicates the proportion of samples that are correctly predicted as positive among all the samples predicted as positive. Recall represents the proportion of samples that are correctly predicted as positive among all the positive samples. F1-score is the harmonic mean of precision and recall.

To better explain these metrics, it is necessary to first explain the concepts related to *TP*, *TN*, *FP*, and *FN*. *TP* means that the sample is predicted as positive and is actually positive. *TN* means that the sample is predicted as negative and is actually negative. *FP* means that the sample is predicted as positive but is actually negative. *FN* means that the sample is predicted as negative but is actually positive.

(14) $$accuracy=\frac{TP+TN}{TP+TN+FN+FP}$$

(15) $$precision=\frac{TP}{TP+FP}$$

(16) $$recall=\frac{TP}{TP+FN}$$

(17) $$F1-Score=\frac{2}{\frac{1}{precision}+\frac{1}{recall}}=2\times \frac{precision\times recall}{precision+recall}$$

In the comparative analysis of classification accuracy, since the F1-score, which is the harmonic mean of precision and recall, can indirectly reflect precision and recall, we chose the F1-score and accuracy as metrics for analysis.

As can be seen from Figure 5, the method proposed in this paper has the highest precision. Therefore, it can be concluded that adding the penalty term, long short-term memory network (LSTM) and central loss term to the model all help to improve the precision. Specifically, adding the penalty term, LSTM and central loss term has increased the precision of the original model by 2.05%, 0.70% and 0.30% respectively. Using the same method, it can be observed that compared with the original model, the models with the penalty term, central loss term and LSTM added have improved the F1-score by 2.16%, 0.71% and 0.20% respectively.

Consequently, in terms of the comprehensive improvement effect, adding the penalty term has the greatest improvement on similarity and classification precision, followed by adding the long short-term network, and finally by adding the central loss term. However, the improvement of the model brought by these three elements all exceeds the performance of the model without adding any of them. Therefore, it can be considered that simultaneously introducing the penalty term, long short-term network and central loss term can better learn the feature distribution of time series data, and then detect anomalies.

### 4.4. Comparative Experiment

#### 4.4.1. Similarity Analysis

To validate the quality of the generated data in the industrial fault domain, the trained generative model is used to generate ten-category data in the basic dataset (CWRU), thereby obtaining the fault data generated by the improved time-series generation method.

**Figure 6:**
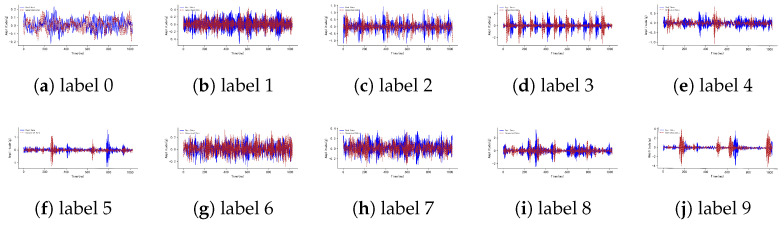
Waveform Comparison Diagram Between Generated Data and Real Data for Labels 0 to 9 Based on the Basic Dataset.

Firstly, from a qualitative perspective, Figure 6 shows the time-domain waveform comparison diagrams between the generated data and the real data of the basic dataset (CWRU). In the waveform comparison of each category, the blue solid line represents the real data, and the red dashed line represents the data generated by the proposed method.

From Figure 6, it can be clearly observed that the generated data maintains a high degree of consistency with the real data in terms of waveform shape, amplitude variation, periodicity, and local details. Furthermore, the generated data not only reproduces the differences between categories but also successfully simulates the local detailed features of the real data, such as instantaneous peaks, demonstrating the proposed method’s strong data reproduction capability.

Overall, the generated data shows a high degree of consistency with the real data in the waveform comparison across various categories, validating the accuracy and reliability of the method in reproducing real data for industrial fault diagnosis. Therefore, we can conclude that the waveforms of the generated data and real data are generally similar. This indicates that the generative model used in the industrial fault diagnosis application scenario is capable of capturing the features of the data and generating waveforms similar to those of the real data, thereby providing high-quality data for fault diagnosis tasks.

In order to analyze the similarity of the generated data more comprehensively, a quantitative analysis is carried out. This paper evaluates the quality of the generated data through Euclidean distance and Wasserstein distance.

**Figure 7:**
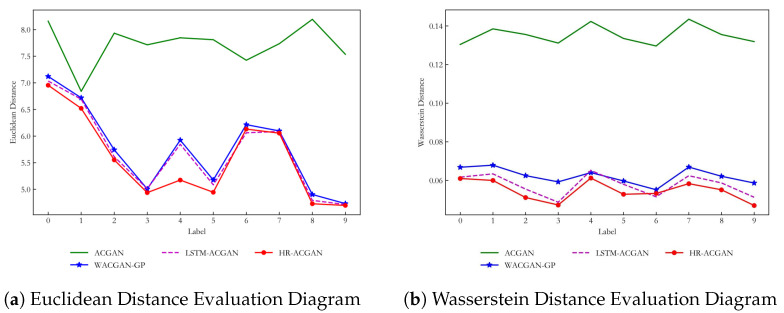
Evaluation Charts of Euclidean Distance and Wasserstein Distance Between Real Data and Generated Data Under Different Generation Methods of the Basic Dataset.

First, with regard to the basic dataset (CWRU), its Euclidean distance will be calculated. As shown in Equation (12), the Euclidean distance does not take into account the gap between the ranges of values of different samples. If the range of values for the different sample sets is excessive, the Euclidean distance will change dramatically in the event of a small change in the larger range of sample sets. This can make the metric less reasonable. Therefore, the normalized dataset is selected as test dataset. We will compare the Euclidean distance of the data generated by ACGAN, WACGAN-GP and LSTM-ACGAN models with the model proposed in this paper. The ACGAN model is an improvement of the GAN model by adding an auxiliary classifier to the discriminator. This makes it possible to both determine whether the data is generated or real and to classify the samples. Therefore, the loss function of this model includes the adversarial loss and classification loss [33,34]. The WACGAN-GP model aims to solve the problems of training instability and pattern collapse in the traditional GAN model. It measures the difference by calculating the Euclidean distance between the real and generated data distributions [35,36]. The LSTM-ACGAN model, on the other hand, combines ACGAN and Long Short-Term Memory (LSTM) networks by introducing LSTM networks in the discriminator. It is able to capture the temporal dependencies of the data better than traditional models. It generates realistic time-series data, thus improving the quality of the generated data [37]. Under the basic dataset, the evaluation results of the Euclidean distance of various generated data by different models are shown in Table 1. In order to provide a more intuitive view of the evaluation results, Euclidean distance evaluation diagram between real data and generated data from different generation methods is shown in Figure 7a.

From the average values in Table 1, the HR-ACGAN method has the highest data similarity among the four models. This is because it considers both the temporal characteristics and the issue of small differences between the categories of the real data. The LSTM-ACGAN method ranks second only to the method proposed in this paper in terms of performance. Although this method considers temporal properties, its temporal property learning ability and accuracy are not as good as the model proposed in this paper. Therefore, the performance is slightly worse. Next is the WACGAN-GP method. This method does not perform as well as LSTM-ACGAN because it does not take into account the timing characteristics and the variability between the data categories. The worst performer is the ACGAN model. This is because it does not take into account both the temporal properties and the low variability between the various categories of the real data. Also, its training stability and convergence are not as stable as WACGAN-GP.

It can be seen from Figure 7a that the distance between the generated data from the ACGAN method and real data is the largest compared with the other methods. This indicates that the generation ability of the ACGAN method is the worst. The comprehensive performance of the proposed HR-ACGAN method is the best. Although the distance for label 6 is slightly higher than the LSTM-ACGAN method, the proposed method outperforms the other methods in the other categories. This proves that the proposed method generates data with high similarity and validates the effectiveness of the proposed method.

Next, we will compare the Wasserstein distance of the data generated by the ACGAN, WACGAN-GP, and LSTM-ACGAN models with the model proposed in this paper. The evaluation results of Wasserstein distance for the different types of generated data from the various models are shown in Table 1. For a more intuitive observation, the Wasserstein distance evaluation graph for the different types of generated data from the various models is presented in Figure 7b.

From Figure 7b, the following conclusions can be drawn: Among all methods, the Wasserstein distance of ACGAN is consistently the highest, indicating that the quality of its generated data is relatively poor and that it exhibits the greatest difference from the real data distribution. This is because ACGAN does not incorporate Wasserstein distance as an optimization objective during training, whereas other methods utilize this distance during training. In contrast, HR-ACGAN achieves the lowest Wasserstein distance under most labels, demonstrating the best generation performance and the closest match to the real data distribution. This is attributed to the multiple improvements in HR-ACGAN compared to other methods, which significantly enhance the diversity and quality of the generated data. Combining Figure 7, it can be observed that HR-ACGAN performs excellently in both distribution matching and feature space approximation, fully demonstrating its advantages in data generation tasks for industrial fault diagnosis.

Therefore, we can conclude that, both qualitatively and quantitatively, the proposed method outperforms other methods in the application of industrial fault diagnosis, with the best overall performance. This method is capable of generating data with high similarity.

#### 4.4.2. Analysis of Classification Accuracy and Scalability

The similarity analysis has proved that the method proposed in this paper can generate data with relatively high similarity. To further validate the scalability of the model in the field of industrial fault diagnosis and to analyze the model’s classification accuracy, we selected the University of Connecticut Gear Fault Dataset (GFD) as an extended dataset on the basis of the basic dataset CWRU to jointly conduct the test on the analysis accuracy.

For the basic dataset (CWRU), in order to test the classification accuracy after expansion, the dataset was first expanded by increasing the number of samples of each type of fault data in the imbalanced sample set of the basic dataset to 1500. The imbalanced sample set of the basic dataset was expanded using the data generated by different methods, and the following expanded datasets were obtained: The data generated by the ACGAN method constituted the ACGAN-basic dataset balanced sample, the data generated by the WACGAN-GP method constituted the WACGAN-GP-basic dataset balanced sample, the data generated by the LSTM-ACGAN method constituted the LSTM-ACGAN-basic dataset balanced sample, and the data generated by the HR-ACGAN method proposed in this paper constituted the HR-ACGAN-basic dataset balanced sample. The specific details of each expanded dataset can be found in Table A5 of Appendix A.

Using the same method, the imbalanced sample set of the extended dataset was expanded by the above four methods, and the following expanded datasets were obtained: The data generated by the ACGAN method constituted the ACGAN-extended dataset balanced sample, the data generated by the WACGAN-GP method constituted the WACGAN-GP-extended dataset balanced sample, the data generated by the LSTM-ACGAN method constituted the LSTM-ACGAN-extended dataset balanced sample, and the data generated by the HR-ACGAN method proposed in this paper constituted the HR-ACGAN-extended dataset balanced sample.

**Figure 8:**
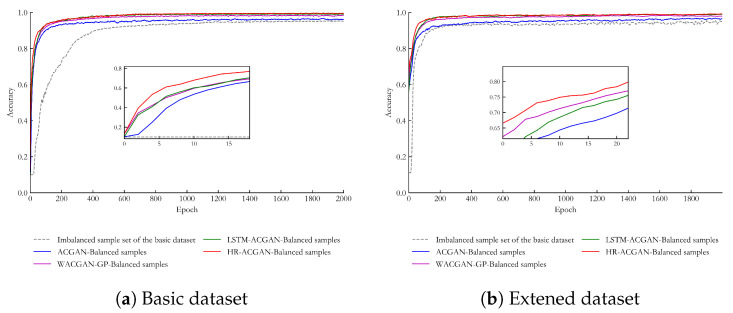
Comparison Diagram of Classification Accuracy on Different Datasets.

The 1D-CNN model was selected as the classification model, and its network parameter settings were as follows: the training batch size was 512, the learning rate was 0.0001, and the Adam algorithm was chosen as the optimizer. The structure of the 1D-CNN model is shown in Table A6 of Appendix A. The imbalanced sample sets of the basic dataset and the extended dataset, the ACGAN-balanced sample dataset, the WACGAN-GP-balanced sample dataset, the LSTM-ACGAN-balanced sample dataset, and the HR-ACGAN-balanced sample dataset were used respectively to train the 1D-CNN model with the same network structure. Then, the test set data was used for testing, and the classification accuracy comparison diagrams of the basic dataset and the extended dataset were obtained respectively, as shown in Figure 8.

It can be seen from Figure 8a that the convergence speeds of the classification accuracies of the other datasets are much faster than that of the imbalanced sample set of the basic dataset. This is because there is a serious sample imbalance problem in the imbalanced sample set of the basic dataset, while the other datasets have become balanced datasets after expansion, so their convergence speeds are relatively faster.

Meanwhile, among these datasets, the classification accuracy of the HR-ACGAN-basic dataset balanced sample dataset ranks first. Next is the LSTM-ACGAN-basic dataset balanced sample dataset, whose accuracy is slightly lower than that of the HR-ACGAN-basic dataset balanced sample dataset. Then comes the WACGAN-GP-basic dataset balanced sample dataset, followed by the ACGAN-basic dataset balanced sample dataset, and the imbalanced sample set of the basic dataset has the lowest accuracy. This indicates that, compared with the other generation methods, after expanding the dataset with the data generated by the proposed HR-ACGAN method, higher classification accuracy can be achieved.

It can be seen from Figure 8b that on the extended dataset, the classification accuracies of various methods still maintain a similar ranking to that of the basic dataset, but the overall accuracies are all lower than those of the basic dataset. This may be because the extended dataset is more complex and poses greater challenges to the classification models. Generally speaking, although the extended dataset is rather complex, the dataset expanded by the HR-ACGAN method can still overcome the impact of data distribution complexity to a certain extent. Therefore, we can draw the conclusion that on the extended dataset (GFD), the model also has a good classification ability, and the model has good extensibility and adaptability. This indicates that the proposed method can effectively adapt to different industrial fault scenarios and maintain high performance in practical applications.

**Figure 9:**
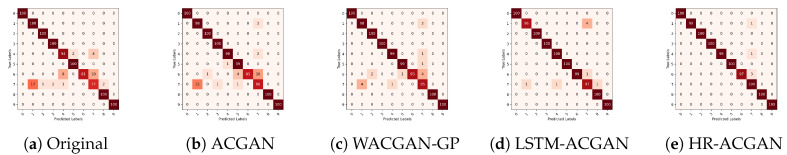
Confusion Matrices of Samples Generated by Different Methods under the Basic Dataset.

**Figure 10:**
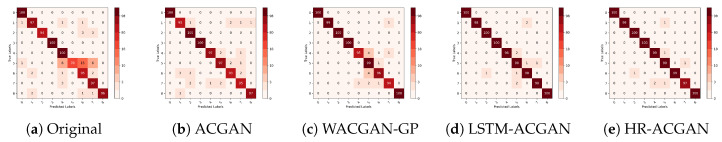
Confusion Matrices of Samples Gnerated by Different Methods under the Extended Dataset.

Furthermore, in order to evaluate the classification effect of each type of data, we drew confusion matrices for the classification results of the 1D-CNN model trained with the datasets. The confusion matrix is an important tool for evaluating the performance of classification models, especially in binary classification and multi-classification problems. It displays the classification results of the classification model on the test dataset in the form of a matrix, including both the correct and incorrect classification cases. Figure 9 and Figure 10 show the confusion matrices of the imbalanced sample sets of the basic dataset and the extended dataset, the ACGAN-balanced sample dataset, the WACGAN-GP-balanced sample dataset, the LSTM-ACGAN-balanced sample dataset, and the HR-ACGAN-balanced sample dataset, respectively.

It can be seen from Figure 9a that the accuracies of label 4, label 6, and label 7 are the lowest, and they are the most likely to be misclassified as other labels. This is because the imbalanced sample set of the basic dataset is an imbalanced dataset in which the number of data in label 4, label 6, and label 7 is less than 200, respectively. As a result, the 1D-CNN classifier is unable to capture sufficient feature information.

Meanwhile, it can also be observed that the accuracies of the confusion matrices of the other datasets have improved to some extent. This is because the other datasets are balanced datasets with generated data added. In particular, for label 4, there is almost no misclassification. For label 6 in the HR-ACGAN-basic dataset balanced sample dataset, the accuracy is the highest, slightly higher than that of the WACGAN-GP-basic dataset balanced sample dataset. The accuracies of the ACGAN-basic dataset balanced sample dataset and the LSTM-ACGAN-basic dataset balanced sample dataset are the same, which are lower than that of the WACGAN-GP-basic dataset balanced sample dataset but higher than that of the imbalanced sample set of the basic dataset. For label 7 in the HR-ACGAN-basic dataset balanced sample dataset, the accuracy is also the highest, slightly higher than that of the LSTM-ACGAN-basic dataset balanced sample dataset and much higher than those of the other datasets. This is because after introducing the improved LSTM network, the generative model can grasp the characteristics of time series data more accurately. Regardless of which category it is, the classification accuracy of the generated data by the proposed method is always the best, indicating that the generated data have good classification performance.

Compared to the basic dataset, the confusion matrices for the extended dataset (Figure 10) demonstrate that the overall performance of various methods has improved, particularly in the classification accuracy of LSTM-ACGAN and HR-ACGAN. Moreover, similar to the basic dataset, HR-ACGAN continues to exhibit the best performance, showcasing its strong sample generation and classification capabilities. This leads to the conclusion that the model possesses excellent scalability and applicability, enabling it to achieve outstanding classification results on the extended dataset (GFD).

The evaluation metrics of the imbalanced sample set of the basic dataset and various expanded datasets are shown in Table 2. As can be seen from Table 2, the recall rates of label 6 and label 7 are extremely low, only around 0.8, and their F1-scores are also below 0.9, indicating that the classification performance for these two types of faults is poor. This is because the imbalanced sample set of the basic dataset is the original imbalanced dataset, and label 6 and label 7 are minority classes, so the classification performance is relatively poor. Meanwhile, the precision, recall rate, and F1-score of label 4 are also relatively low, which is also caused by the small amount of data in label 4. Moreover, according to the confusion matrix, it can be observed that label 6 will be misclassified as label 4 or label 7, which is also the reason why the evaluation metrics of label 4 are relatively low. We also found that although the number of samples in label 1 is relatively large, its precision and F1-score are still relatively low. Similarly, by observing the confusion matrix, it can be seen that this is because label 7 is misclassified as label 1. If sufficient data information of label 7 is added, the classification performance will be improved and the probability of misclassification will be reduced.

The evaluation metrics of the other expanded datasets are shown in Table 2. Compared with the original dataset, the values of the expanded dataset on all four metrics have been improved. Among them, the accuracy of the HR-ACGAN-basic dataset balanced sample dataset is the highest, slightly higher than that of the LSTM-ACGAN-basic dataset balanced sample dataset. Next comes the WACGAN-GP-basic dataset balanced sample dataset, and finally the ACGAN-basic dataset balanced sample dataset, all of which are significantly higher than the basic dataset imbalanced sample set. Compared with the basic dataset imbalanced sample set, both the precision and F1-score of the ACGAN-basic dataset balanced sample dataset on the minority classes have been improved, but its performance on label 7 is not good. This is because, based on the analysis of the confusion matrix, label 7 and label 1 appear to be confused when classifying categories, which is due to the fact that the performance of the generative model still needs to be improved. The F1-scores of the other classes are all higher than 0.9.

From Table 2, it can be observed that the other datasets have achieved significant improvements in all metrics, and the F1-score of each label are all above 0.9. This is because they take into account the model stability and the temporal characteristics of the data, which proves that WACGAN-GP, LSTM-ACGAN, and the proposed method all have relatively high classification performance. Among them, the comprehensive performance of the HR-ACGAN-basic dataset balanced sample dataset is the highest. This is because the proposed model takes into account both stability and time series characteristics and also limits the range of the generated data at the same time, indicating that the proposed method can not only conduct effective modeling but also enable the expanded data to obtain better classification performance.

The evaluation metrics for the imbalanced sample set of the extended dataset and the various augmented datasets are shown in Table 3. Upon comparison, the fluctuation ranges of the metrics (accuracy, recall, F1-score) for the basic dataset and the extended dataset are similar when dealing with the imbalanced samples, although the extended dataset shows slightly lower values for certain categories. After generating balanced datasets using the various methods, the metrics (accuracy, recall, F1-score) for both the basic dataset and the extended dataset have improved and become more similar. Notably, after the extended dataset was augmented using ACGAN, the F1-score became more balanced, with values that were previously around 0.8 improving to over 0.9. However, the accuracy of the extended dataset remains generally lower than that of the basic dataset. This may be due to the increased complexity of the extended dataset, which impacts model performance, although the various methods can somewhat alleviate this effect. Therefore, we can conclude that the model not only demonstrates strong classification ability but also exhibits excellent scalability.

In order to further test the classification performance, other widely used classification models are also used to test the above datasets, including the LSTM, ELM, and SVM models. LSTM is a special type of recurrent neural network (RNN) dedicated to the processing and prediction of time series data. It controls the flow of information through memory cells and gating mechanisms [38]. ELM is a learning algorithm for single hidden layer feedforward neural networks (SLFNs). With an extremely simplified training process, ELM has the advantage of fast training speed and high generalization ability. Therefore, it is used for classification, regression, and feature learning on small sample datasets [39]. SVM is a supervised learning model for classification and regression analysis. It performs well with high-dimensional data and is especially suitable for small samples and complex non-linear data [40]. These algorithms are representative and effective in handling time series data and small sample datasets, respectively. Therefore, in the experiments within this paper, these algorithms are used to test the above datasets to further evaluate the classification performance.

The classification accuracy of the basic dataset and the datasets expanded by the generated data from the different methods is shown in Table 4. From Table 4, it can be seen that the variation in the classification accuracy of all the classification models is basically the same as the 1D-CNN model. The proposed HR-ACGAN method has the highest classification accuracy.

The classification accuracy of the expanded dataset and the datasets augmented by the various methods is shown in Table 5. As can be seen from Table 5, the classification accuracy trends for the expanded dataset under the four classification models are consistent with those of the basic dataset. Specifically, the proposed HR-ACGAN method achieves the highest classification accuracy, followed by LSTM-ACGAN, while the original dataset, due to the data imbalance issue, has the lowest classification accuracy. Therefore, we can conclude that the proposed method not only achieves the highest classification accuracy on the base dataset but also performs well on the expanded dataset (GFD), demonstrating high classification accuracy.

Through the similarity and classification accuracy tests and analyses above, it can be seen that the proposed HR-ACGAN method can generate data that is similar to real data and effectively augment the CWRU dataset and GFD dataset in the industrial fault domain, thereby improving classification accuracy. The test results fully validate the effectiveness and superiority of the proposed method in industrial fault diagnosis, providing reliable data support for fault detection and diagnosis tasks.

## 5. Conclusions

Most of the existing data generation methods do not take into account the temporal dynamics of time series. This results in generative models that may not be efficient enough to capture the gradual dependencies in the training data. In multi-classification problems, most methods only consider how to constrain the generation of data with specified labels. However, the problem of generating too much overlap between samples is not constrained. In order to solve the above problems, a time series data generation method with high reliability based on ACGAN (HR-ACGAN) is proposed. The proposed method not only considers the temporal characteristics of time series data, but also improves the reliability of the generated data by improving the objective function of the generator. This method is specifically optimized for the small sample problem in the field of industrial fault diagnosis, aiming to generate data that is highly similar to real fault data in order to enhance the performance of fault diagnosis models. A comparative test of different generation methods is carried out by using the basic dataset (CWRU) and the extended dataset (GFD). The results of the similarity test show that the data generated by the proposed method HR-ACGAN has a relatively high similarity with the real data. Both the Euclidean distance and the Wasserstein distance between the generated data and the real data are lower than those of other comparative methods. The classification accuracy test results show that the dataset expanded with the data generated by the proposed method has higher classification accuracy. The overall test results indicate that the proposed method can generate data with higher reliability, significantly improving model performance, and validating the effectiveness and superiority of the method in industrial fault diagnosis. Since the model adopts a 1D-CNN structure, both the generator’s output and the discriminator’s input are one-dimensional data. Therefore, this approach is particularly suitable for handling time-series data, such as in industrial fault diagnosis applications. However, when dealing with tasks involving multiple data features, such as in the financial sector, the method still requires further expansion and optimization to accommodate a broader range of application scenarios.

## Figures and Tables

**Table 1:** Evaluation Results of the Euclidean Distance and Wasserstein Distance Between the Real Data and the Data Generated by Different Generation Methods under the Basic Dataset.

Metrics	Methods	Labels										
		0	1	2	3	4	5	6	7	8	9	Avg.
Euclidean Distance	ACGAN	8.16	6.84	7.93	7.72	7.85	7.81	7.43	7.74	8.19	7.54	7.72
	WACGAN-GP	7.12	6.72	5.74	5.00	5.92	5.18	6.22	6.10	4.90	4.73	5.76
	LSTM-ACGAN	7.03	6.69	5.61	5.01	5.85	5.09	6.06	6.09	4.80	4.72	5.69
	HR-ACGAN	6.96	6.52	5.55	4.94	5.17	4.95	6.13	6.06	4.73	4.70	5.57
Wasserstein Distance	ACGAN	0.130	0.138	0.136	0.131	0.142	0.134	0.130	0.143	0.136	0.132	0.135
	WACGAN-GP	0.067	0.068	0.062	0.059	0.064	0.060	0.055	0.067	0.062	0.059	0.062
	LSTM-ACGAN	0.062	0.063	0.055	0.049	0.065	0.058	0.051	0.062	0.059	0.051	0.058
	HR-ACGAN	0.061	0.060	0.051	0.047	0.061	0.053	0.053	0.058	0.055	0.047	0.055

**Table 2:** Evaluation Metrics of the Imbalanced Sample Set of the Basic Dataset and Various Expanded Datasets.

Different Datasets	Metrics	Labels									
		0	1	2	3	4	5	6	7	8	9
Imbalanced sample	Precision	1.00	0.86	0.99	0.98	0.90	0.98	1.00	0.85	0.98	1.00
	Recall	1.00	1.00	1.00	1.00	0.94	1.00	0.81	0.77	1.00	1.00
	F1-score	1.00	0.92	0.99	0.99	0.92	0.99	0.89	0.81	0.99	1.00
	Accuracy					0.952					
ACGAN-balanced sample	Precision	1.00	0.89	0.99	0.99	0.99	0.95	1.00	0.86	1.00	1.00
	Recall	1.00	0.98	1.00	1.00	0.98	0.99	0.85	0.86	1.00	1.00
	F1-score	1.00	0.93	0.99	0.99	0.98	0.97	0.91	0.85	1.00	1.00
	Accuracy					0.966					
WACGAN-GP-balanced sample	Precision	1.00	0.96	0.98	1.00	0.99	0.99	1.00	0.92	1.00	1.00
	Recall	1.00	0.98	1.00	1.00	0.99	0.99	0.93	0.95	1.00	1.00
	F1-score	1.00	0.97	0.99	1.00	0.99	0.99	0.96	0.94	1.00	1.00
	Accuracy					0.984					
LSTM-ACGAN-balanced sample	Precision	1.00	0.99	1.00	1.00	0.99	1.00	1.00	0.95	0.99	1.00
	Recall	1.00	0.96	1.00	1.00	1.00	1.00	0.99	0.97	0.99	1.00
	F1-score	1.00	0.97	1.00	1.00	0.99	1.00	0.99	0.96	0.99	1.00
	Accuracy					0.992					
HR-ACGAN-balanced sample	Precision	1.00	1.00	1.00	1.00	1.00	1.00	1.00	0.95	1.00	1.00
	Recall	1.00	0.99	1.00	1.00	0.99	1.00	0.97	1.00	1.00	1.00
	F1-score	1.00	0.99	1.00	1.00	0.99	1.00	0.98	0.98	1.00	1.00
	Accuracy					0.995					

**Table 3:** Evaluation Metrics of the Imbalanced Sample Set of the Extended Dataset and Various Expanded Datasets.

Different Datasets	Metrics	Labels								
		0	1	2	3	4	5	6	7	8
Imbalanced sample	Precision	0.98	0.96	1.00	1.00	0.885	1.00	0.826	0.890	1.00
	Recall	1.00	0.97	0.94	1.00	1.00	0.70	0.95	0.97	0.96
	F1-score	0.99	0.96	0.97	1.00	0.94	0.82	0.88	0.93	0.98
	Accuracy					0.943				
ACGAN-balanced sample	Precision	1.00	0.94	0.952	1.00	0.99	0.96	0.95	0.95	0.99
	Recall	1.00	0.95	1.00	1.00	0.97	0.97	0.93	0.95	0.97
	F1-score	1.00	0.95	0.98	1.00	0.98	0.96	0.94	0.95	0.98
	Accuracy					0.971				
WACGAN-GP-balanced sample	Precision	1.00	1.00	1.00	1.00	0.97	0.90	0.98	0.98	1.00
	Recall	1.00	0.99	1.00	1.00	0.95	0.99	0.96	0.94	1.00
	F1-score	1.00	0.99	1.00	1.00	0.96	0.94	0.97	0.96	1.00
	Accuracy					0.981				
LSTM-ACGAN-balanced sample	Precision	1.00	1.00	0.99	1.00	1.00	0.95	0.97	0.99	1.00
	Recall	1.00	0.98	1.00	1.00	0.98	0.98	0.98	0.98	1.00
	F1-score	1.00	0.99	0.99	1.00	0.99	0.97	0.97	0.98	1.00
	Accuracy					0.988				
HR-ACGAN-balanced sample	Precision	1.00	1.00	0.99	1.00	0.98	0.97	1.00	0.99	1.00
	Recall	1.00	0.99	1.00	1.00	0.99	0.99	0.99	0.97	1.00
	F1-score	1.00	0.99	0.99	1.00	0.98	0.98	0.99	0.98	1.00
	Accuracy					0.992				

**Table 4:** The Classification Accuracy of Mixed Data of Different Testing Models Based on the Basic Dataset.

Datasets\Models	Original Dataset	ACGAN	WACGAN-GP	LSTM-ACGAN	HR-ACGAN
1D-CNN	95.2%	96.6%	98.4%	99.2%	99.5%
LSTM	94.9%	95.8%	98.0%	98.8%	99.2%
ELM	95.1%	96.1%	97.8%	98.1%	98.9%
SVM	94.6%	95.3%	97.5%	97.9%	98.5%

**Table 5:** The Classification Accuracy of Mixed Data of Different Testing Models Based on the Extended Dataset.

Datasets\Models	Original Dataset	ACGAN	WACGAN-GP	LSTM-ACGAN	HR-ACGAN
1D-CNN	94.3%	97.1%	98.1%	98.9%	99.2%
LSTM	93.8%	96.1%	97.5%	98.4%	98.3%
ELM	94.2%	96.3%	97.1%	97.5%	97.9%
SVM	92.4%	93.2%	96.7%	97.9%	98.1%

## Data Availability

The data used to support the findings of this study are available from the corresponding author upon request.

## Appendix A

**Table A1:** Description of the Selected Basic Dataset.

Label	Fault Type	Fault Diameter/inch	Load/hp	Number of Samples
0	Normal	0	0∼3	1600
1	Ball	0.007	0∼3	400
2	Inner Race	0.007	0∼3	400
3	Outer Race	0.007	0∼3	400
4	Ball	0.014	0∼3	400
5	Inner Race	0.014	0∼3	400
6	Outer Race	0.014	0∼3	400
7	Ball	0.021	0∼3	400
8	Inner Race	0.021	0∼3	400
9	Outer Race	0.021	0∼3	400

**Table A2:** Size of Experimental Datasets under the Basic Dataset.

Label	Imbalanced Set	Training Set	Testing Set
0	1500	150	100
1	249	150	100
2	300	150	100
3	292	150	100
4	195	150	100
5	212	150	100
6	150	150	100
7	170	150	100
8	271	150	100
9	232	150	100

**Table A3:** Structure of Generator Model.

Network Layer	Convolution Kernel	Channel Numbers	Step Length	Activation Function
Input				
Upsampling (scale_factor = 2)				
Convld	5	128	1	ReLU
Convld	5	128	1	ReLU
BatchNormld				
Convld	5	128	1	ReLU
Convld	5	128	1	ReLU
BatchNormld				
Upsampling (scale_factor = 2)				
Convld	5	64	1	ReLU
Convld	5	64	1	ReLU
BatchNormld				
Convld	5	64	1	ReLU
Convld	5	64	1	ReLU
BatchNormld				
Upsampling (scale_factor = 2)				
Convld	5	16	1	ReLU
Convld	5	16	1	ReLU
BatchNormld				
Convld	5	16	1	ReLU
Convld	5	16	1	ReLU
BatchNormld				
Convld	5	1	1	Tanh

**Table A4:** Structure of Discriminator Model.

Network Layer	Convolution Kernel	Channel Numbers	Step Length	Activation Function
Input				
Convld	5	64	2	LeakyReLU (0.2)
Convld	5	64	1	LeakyReLU (0.2)
Dropput (0.5)				
Convld	5	64	2	LeakyReLU (0.2)
Convld	5	64	1	LeakyReLU (0.2)
Dropput (0.5)				
MaxPoolld	5		2	
Convld	5	128	2	LeakyReLU (0.2)
Convld	5	128	1	LeakyReLU (0.2)
Dropput (0.5)				
Convld	5	128	2	LeakyReLU (0.2)
Convld	5	128	1	LeakyReLU (0.2)
Dropput (0.5)				
MaxPoolld	5		2	
Convld	5	256	2	LeakyReLU (0.2)
Convld	5	256	1	LeakyReLU (0.2)
Dropput (0.5)				
Convld	5	256	2	LeakyReLU (0.2)
Convld	5	256	1	LeakyReLU (0.2)
Dropput (0.5)				
AvgPolld	5		2	
BiLSTM (4)				
Linear1				
Linear2				

**Table A5:** Situation of Expanded Datasets Based on the Basic Dataset.

Label	Testing Set	Imbalanced Set	ACGAN	WACGAN-GP	LSTM-ACGAN	HR-ACGAN
0	100	1500	1500	1500	1500	1500
1	100	249	249 (1251)	249 (1251)	249 (1251)	249 (1251)
2	100	300	300 (1200)	300 (1200)	300 (1200)	300 (1200)
3	100	292	292 (1208)	292 (1208)	292 (1208)	292 (1208)
4	100	195	195 (1305)	195 (1305)	195 (1305)	195 (1305)
5	100	212	212 (1288)	212 (1288)	212 (1288)	212 (1288)
6	100	150	150 (1350)	150 (1350)	150 (1350)	150 (1350)
7	100	170	170 (1330)	170 (1330)	170 (1330)	170 (1330)
8	100	271	271 (1229)	271 (1229)	271 (1229)	271 (1229)
9	100	232	232 (1268)	232 (1268)	232 (1268)	232 (1268)

**Table A6:** Structure of 1D-CNN Model.

Network Layer	Convolution Kernel	Channel Numbers	Step Length	Activation Function	Output
Input					1 × 1024
Convld	3	16	2	ReLU	16 × 512
MaxPoolld			2		16 × 256
Convld	3	64	2	ReLU	64 × 128
MaxPoolld			2		64 × 64
Convld	3	256	2	ReLU	256 × 32
MaxPoolld			2		256 × 16
Convld	3	512	2	ReLU	512 × 8
AvgPoolld (8)					512 × 1
Flatten					512 × 512
Linear					512 × 10
SoftMax					512 × 10
